# What is the impact of intravitreal injection of conbercept on neovascular glaucoma patients: a prospective, interventional case series study

**DOI:** 10.1186/s12886-019-1138-6

**Published:** 2019-06-11

**Authors:** Liukun Shi, Jin Yang, Jinyong Lin

**Affiliations:** Tianjin Eye Hospital, Tianjin Key Lab of Ophthalmology and Visual Science, Clinical College of Ophthalmology, Tianjin Medical University, No.4 Gansu Rd, Heping District, Tianjin, 300020 China

**Keywords:** Neovascular glaucoma, Conbercept, Anti-vascular endothelial growth factor, Trabeculectomy

## Abstract

**Background:**

The aim of the present study was to evaluate the efficacy and safety of intravitreal conbercept combined with trabeculectomy and panretinal photocoagulation for neovascular glaucoma (NVG).

**Methods:**

Fifty patients (54 eyes) with NVG were included in this prospective study. Fifty-two eyes initially underwent intravitreal conbercept (0.5 mg/0.05 ml) treatment followed by trabeculectomy and panretinal photocoagulation. Preoperative and postoperative best-corrected visual acuity (BCVA), intraocular pressure (IOP), the number of antiglaucoma medications, and surgical complications were recorded. The levels of VEGF-A, TGF-β_1_ and PLGF in aqueous humour samples collected during surgery were measured by enzyme-linked immunosorbent assay (ELISA). Light microscopy and transmission electron microscopy were used to observe the surgically excised trabecular tissue; enucleation was performed in 2 eyes, and light microscopy was used as the histopathological control.

**Results:**

The follow-up period after trabeculectomy was 1 year. Of the 52 eyes, 39 completed 1 year of follow-up, and 13 were lost to follow-up. Recurrence of iris neovascularization was observed in 5 eyes, 9 had hyphema, 16 had filter-bled scarring, and no eye had complications attributable to the drug. The mean IOP was reduced from 48.1 ± 14.2 to 23.2 ± 8.7 mmHg, and the mean number of antiglaucoma medications used decreased from 3.0 (3.0, 4.0) to 1.0 (0.0, 1.0) after 1 year (both *P* < 0.05). The complete success rate was 76.9, 76.9, 71.0, 51.6, and 32.3% at 1 week, 1 month, 3 months, 6 months and 12 months, respectively, when the cut-off IOP was 18 mmHg. After patients underwent intravitreal injection, the concentrations of VEGF-A and TGF-β_1_ in the aqueous humour in NVG patients decreased from 168.8 ± 13.4 and 159.6 ± 15.4 pg/ml to 160.2 ± 7.6 and 151.9 ± 2.3 pg/ml, respectively (both *P* < 0.05). Light microscopy revealed neovascularization regression in the iris in specimens treated with intravitreal conbercept. Electron microscopy revealed trabecular endothelial cell degeneration in the conbercept-treated specimens.

**Conclusions:**

Our initial findings suggest that intravitreal conbercept is an effective treatment for managing NVG that has fewer short-term postoperative complications.

**Trial registration:**

Current Controlled Trials ChiCTR1800019918, 8 December 2018, retrospectively registered.

**Electronic supplementary material:**

The online version of this article (10.1186/s12886-019-1138-6) contains supplementary material, which is available to authorized users.

## Background

Neovascular glaucoma (NVG) is a refractory glaucoma caused by retinal ischaemic disease, such as central retinal vein occlusion (CRVO), diabetic retinopathy (DR), and ocular ischaemic syndrome, and is characterized by neovascularization of the iris and anterior chamber angle and high intraocular pressure (IOP) [1]. Clinical treatments for NVG include reducing IOP and treating the primary disease. Using anti-glaucoma drugs or panretinal photocoagulation (PRP) may be effective in the early rubeosis iridis and open-angle glaucoma stages. However, in the angle-closure glaucoma stage of NVG, IOP increases rapidly because proliferative fibrovascular membranes cause angle closure; thus, glaucoma-filtering surgery is effective, but its success rate is poor due to iris and anterior chamber angle neovascularization.

Vascular endothelial growth factor (VEGF) is synthesized because of retinal ischaemia and hypoxia [2]. PRP does not rapidly regress the neovascularization of the iris and angle and is difficult to perform in eyes with media opacities due to high IOP. Intravitreal anti-VEGF agents have been confirmed to decrease the concentration of VEGF in the aqueous humour and regress iris neovascularization. Therefore, the use of anti-VEGF agents before trabeculectomy can reduce intraoperative and postoperative complications [2–4]. PRP can improve retinal ischaemia status, thus reducing the release of VEGF. According to previous studies, intravitreal injection of anti-VEGF agents, such as bevacizumab and ranibizumab combined with glaucoma-filtering surgery, and PRP has been shown to rapidly regress iris neovascularization and to effectively reduce IOP [3, 4].

Recently, a new anti-VEGF agent, conbercept (KH902, Chengdu Kang Hong biotech Co., Sichuan, Chengdu), has been shown to quickly regress choroidal neovascularization in age-related macular disease in a clinical setting [5], but the effect of conbercept on iris neovascularization is unknown. This study was performed to assess the efficacy and safety of conbercept for the treatment of NVG in 54 eyes of 50 NVG patients and explore the relationships between NVG and a variety of factors, including VEGF-A, transforming growth factor-β1 (TGF-β1), and placenta growth factor (PLGF) and their usefulness as targets for NVG.

## Methods

We performed a study of a consecutive series of 54 eyes of 50 patients who were diagnosed with NVG in Tianjin Eye Hospital from May 2015 to March 2017. Fifty-two eyes of 48 patients received intravitreal conbercept combined with trabeculectomy. The other 2 eyes had absolute glaucoma with unbearable pain and were treated with enucleation to identify iris neovascularization by light microscopy; these eyes were used as a control group.

The inclusion criteria were (1) NVG patients whose underlying diseases were DR, CRVO, carotid artery stenosis or occlusive disease; (2) patients not previously treated with intravitreal anti-VEGF agents or PRP; (3) patients who were 18 to 85 years old; (4) patients with a visual acuity ≥ hand motion; (5) patients with an IOP > 21 mmHg (1 mmHg = 0.133 kPa) who were receiving the maximum dose of antiglaucoma medications; (6) patients with iris or angle neovascularization; and (7) patients with or without pupillary margin pigmented layer eversion. The exclusion criteria were (1) NVG combined with ocular tumours or uveitis, (2) severe cardiovascular or cerebrovascular diseases, (3) patients who recently had active inflammation of the eye, or (4) patients who failed to complete the scheduled follow-ups for various reasons. The study was carried out with the approval of the ethics committee of Tianjin Eye Hospital (No. TJYYLL-2015-15), and all patients provided voluntary informed consent to participate in the study.

A preoperative evaluation included best corrected visual acuity (BCVA), slit-lamp microscopy and gonioscopy examinations, IOP, primary disease classification and other measures. BCVA was converted to the logarithm of the minimum angle of resolution (LogMAR), and IOP was measured with a Goldmann tonometer (AT 900, Haag-Strsit AG, Switzerland, Bern) except in patients with corneal oedema due to high IOP, in whom an Icare handheld rebound tonometer (TA01i, Finland Aike company, Finland, Helsinki) was used. Iris neovascularization grades were scored as follows: grade 1, surface neovascularization of the pupillary zone of the iris involving ≤2 quadrants; grade 2, surface neovascularization of the pupillary zone of the iris involving ≥2 quadrants; grade 3, neovascularization of the ciliary zone of the iris involving 1 to 3 quadrants in addition to the pupillary zone; and grade 4, neovascularization of the ciliary zone of the iris involving ≥3 quadrants [6].

All patients were assessed by one glaucoma specialist. Under sterilization and topical anaesthesia (0.5% proparacaine hydrochloride eye drops), anterior chamber paracentesis was performed in 48 patients (52 eyes) with high IOP and followed by intravitreal injection of 0.05 ml (0.5 mg) of conbercept via a 30G needle at the pars plana (approximately 3.5 mm from the limbus). Then, IOP and light perception were examined. Patients were given topical antibiotics and antiglaucoma medications after surgery.

Trabeculectomy was performed when the neovascularization of the iris surface regressed and anterior chamber inflammation was relieved at an interval of 2 to 7 days. Under peribulbar anaesthesia, a fornix-based conjunctival flap and a half-thickness 4*3 mm square scleral flap were made, and a mitomycin C (MMC) (0.4 mg/ml) or 5-fluorouracil (5-FU) (25 mg/ml)-soaked sponge was placed in the scleral flap for 2–3 min before rinsing thoroughly with 30 ml saline. The trabecular meshwork (2*1 mm) was cut, and peripheral iridectomy was performed. The scleral flap was closed with two 10–0 nylon sutures at its corners, and the conjunctiva was sutured with 10–0 nylon sutures.

Two patients (2 eyes) underwent enucleation under retrobulbar anaesthesia. The bulbar conjunctiva was cut along the limbus corneae, and the conjunctiva was then separated. The tenon capsule and sclera were bluntly dissected to the equator, the extraocular muscles were cut, the optic nerve and soft tissue were removed, the eye was removed, a enucleated eye was implanted in the tenon capsule, the front fascia was closed by pouch suture, the tenon capsule was packed with Vaseline gauze, and the eye socket was placed.

Three weeks or 1 month later, PRP was performed (577 nm yellow light via supra-scan laser light, Quantel, France, Clermont-Ferrand) 2–3 times with 250–350 mv of energy (the appearance of II-III spots was the standard) at an interval time of 0.03 s, a spot diameter of 200 μm, and a total light solidifying point of 1500–2000 points.

The surgically excised trabecular specimens of 26 eyes were immediately immersed in a solution of 10% formalin fixative at room temperature for 24 h, and these specimens were then dehydrated with different concentrations of alcohol (from 70 to 100%) and made transparent via two washes in dimethylbenzene. After the tissues were embedded in paraffin, 5-μm-thick sections were cut, mounted and allowed to dry overnight. Then, these specimens were stained with haematoxylin-eosin and observed with a light microscope (Leica 400B, Leica company, Germany, Solms).

The iris tissues of the other eyes were immersed in 4% glutaraldehyde and 1% osmic acid solution at 4 °C for 4 h and 1 h, respectively, and these specimens were then washed with PBS powder and dehydrated through a gradient of acetone solutions. After the tissues were embedded in 812 resin, the specimens were cut into 60 nm-thick sections and stained with lead citrate and uranyl acetate. Transmission electron microscopy (JEM-1230, JEOL company, Japan, Tokyo) was used to view these sections.

We collected 0.1–0.15 ml aqueous humour samples at the same time as the intravitreal injections and trabeculectomy. These were placed in sterile Eppendorf tubes and rapidly frozen at − 80 °C until used (all samples were obtained at the beginning of surgery to avoid breakdown of the blood-aqueous barrier, which is associated with surgical trauma). The main reagents were human VEGF-A enzyme-linked immunosorbent assay (ELISA) and human TGF-β1 ELISA kit and human PLGF ELISA (China BlueGene Co., China, Shanghai) kits. The concentrations of VEGF-A, TGF-β1 and PLGF in the aqueous humour were determined by the double antibody sandwich ELISA method. A standard curve was set up for ELISA (using a four-parameter logistic curve fit). The desired numbers of coated wells were secured in the holder, and then 100 μL of each standard or sample was added to the appropriate well. Then, 100 μL of PBS (pH 7.0–7.2) was added to the blank control well followed by 50 μL of conjugate to every well but the blank control well. The plates were covered and incubated for 1 h at 37 °C. The plates were washed five times with diluted wash solution (350–400 μL/well/wash) using an auto washer and then dried. Next, 50 μL of substrate A and 50 μL of substrate B was added to each well, and the plates were covered and incubated for 15 min at 37 °C. Then, 50 μL of stop solution was added to each well, and the optical density (O.D.) was immediately determined at 450 nm using a microplate reader (ST-360, Shanghai KEHUA Experimental System Co., Shanghai, China).

The postoperative follow-up period was 1 year. Patients were followed on a schedule (post-injection and at 1 day, 1 week, 1 month, 3 months, 6 months, and 1 year after trabeculectomy). BCVA and IOP were recorded. Slit-lamp microscopy and gonioscopy examination results, the number of anti-glaucoma medications, and intraoperative and postoperative complications were recorded. A previous study showed that the complete success of this surgery was defined as an IOP ≤21 mmHg without any topical ocular hypotensive medication, partial success was defined as an IOP ≤21 mmHg with topical ocular hypotensive medication [7], and surgical failure was defined as IOP > 21 mmHg at 2 consecutive follow-up visits even with anti-glaucoma medication or additional glaucoma surgeries, such as filtration surgery and cyclophotocoagulation, or loss of light perception [8]. However, in these eyes, an IOP of 21 mmHg is still very high, and the cut-off should really be 18 mmHg, with complete and partial success based on cut-off IOP values of 21, 18 and 15 mmHg depending on the time points and estimated to obtain a detailed overview of the exact results.

Statistical analyses were conducted using SPSS 23.0 software (SPSS Inc., Chicago, America). The data on IOP, BCVA and the concentrations of cytokines were confirmed by the W test and were consistent with a normal distribution. Therefore, these results are presented as the mean ± standard deviation (SD). The data on the numbers of antiglaucoma drugs were not consistent with a normal distribution. Therefore, these results are expressed as M (Q1, Q3). Single-effect repeated-measure analysis of variance and Dunnett’s t-test were used to assess differences between BCVA and IOP at different time points. The levels of VEGF-A, TGF-β1 and PLGF were compared by one-way ANOVA and LSD t-tests. The number of antiglaucoma medications was compared across groups by K-W tests of multiple groups of independent samples. The correlation analysis between iris neovascularization and VEGF-A, TGF-β1, and PLGF was completed by Spearman rank correlation coefficients. Kaplan-Meier survival analysis was performed to estimate surgical success. *P* values lower than 0.05 were considered statistically significant.

## Results

This study included 37 males (40 eyes) and 11 females (12 eyes) ranging in age from 18 to 82 (55.9 ± 14.7) years old. Twenty-three eyes had DR, 23 had CRVO, 5 had ocular ischaemic syndrome, and 1 had central retinal artery occlusion. The mean preoperative IOP was 48.1 ± 14.2 mmHg, and the iris neovascularization grades were 4, 3, and 2 in 25, 17 and 10 patients, respectively. At 1 year, visual acuity remained unchanged in 16 eyes, had increased in 16 eyes, and had decreased in 7 eyes. The complete success rates were 76.9, 76.9, 71.0, 51.6, and 32.3% at 1 week, 1 month, 3 months, 6 months and 12 months, respectively, when the cut-off for IOP was 18 mmHg. Twenty-five eyes were considered surgical failures, and among these, after the antiglaucoma medicine was administered and the filter blebs massaged and needed via subconjunctival injection, IOP was decreased in the 20 eyes with an IOP > 18 mmHg at 2 consecutive follow-up visits. The IOP of 5 eyes with previously uncontrolled IOP was controlled by transscleral cyclophotocoagulation (TCP) or trabeculectomy. The data on IOP, BCVA and the number of antiglaucoma medications are shown in Table 1, and the complete and partial success (based on cut-offs at 21, 18 and 15 mmHg at different times) are shown in Table 2.

**Table 1:** IOP, BCVA and the number of antiglaucoma medications for NVG patients

time	eyes	IOP (mmHg)^b^	BCVA (LogMAR)^b^	antiglaucoma medications^c^
baseline	52	48.1 ± 14.2	2.3 ± 1.1	3.0 (3.0, 4.0)
after injection	52	44.3 ± 14.0	2.2 ± 1.1	3.0 (3.0, 4.0)
1 day	52	19.5 ± 10.6^a^	2.2 ± 1.1	0.0 (0.0, 0.0)^a^
1 week	49	16.1 ± 9.3^a^	1.9 ± 1.0	0.0 (0.0, 0.0)^a^
1 month	42	20.2 ± 8.9^a^	2.0 ± 1.1	0.0 (0.0, 0.0)^a^
3 months	40	23.1 ± 10.6^a^	2.0 ± 1.2	0.0 (0.0, 0.8)^a^
6 months	39	25.1 ± 8.6^a^	2.0 ± 1.2	0.0 (0.0, 1.0)^a^
1 year	39	23.2 ± 8.7^a^	2.1 ± 1.2	1.0 (0.0, 1.0)^a^
*F/χ* ^*2*^		878.486	163.157	294.232
*P*		0.000	0.000	0.000

Compared with pre-injection, ^a^*P* < 0.05.^b^:ANOVA, Dunnett-*t* test ^c^:Kruskal-Wallis test

**Table 2:** Success rates at different time points for NVG(%)

	cut off 21 mmHg		cut off 18 mmHg		cut off 15 mmHg	
	complete success	partial success	complete success	partial success	complete success	partial success
1 week	88.5	91.3	76.9	78.3	53.8	65.2
1 month	88.5	86.7	76.9	65.2	47.1	52.2
3 months	82.6	38.6	71.0	30.4	40.4	17.4
6 months	69.9	24.1	51.6	21.7	33.7	13.0
12 months	57.2	14.5	32.3	8.7	8.4	4.3

Kaplan-Meier survival analysis

The differences observed in IOP and BCVA at various follow-up time points were statistically significant (F = 878.486, *p* = 0.000; *F* = 163.157, *p* = 0.000). IOP gradually decreased over time, and IOP was significantly lower after trabeculectomy than at baseline (all *p* = 0.000). BCVA was not significantly higher postoperatively than at baseline (all *p* > 0.05). There was a statistically significant difference at follow-up time points in the number of antiglaucoma medications (*χ*^*2*^ = 294.232, *p* = 0.000), and the number of antiglaucoma medications was significantly lower after trabeculectomy (all *p* = 0.000) (Table 1).

Hyphema occurred in 9 eyes and was absorbed 2–3 days later. Five eyes underwent laser cutting, and 18 eyes underwent filter bleb massage. Sixteen eyes with filter bleb scarring received subconjunctival injection of 5-FU combined with bleb separation. One eye, 2 eyes and 2 eyes had iris neovascularization recurrence and an increased IOP at 2 months, 3 months and 5 months postoperatively, respectively, and IOP decreased in 2 of these eyes after PRP. The other 3 eyes had uncontrolled IOP and received TCP. One eye with uncontrolled IOP received a large dose of antiglaucoma medication, had no light perception, and was treated with TCP, resulting in a normal IOP at 2 months. One eye with high IOP that was ineffectively treated with antiglaucoma drugs received trabeculectomy after 11 months.

The concentrations of VEGF-A and TGF-β1 in aqueous humour samples were 168.8 ± 13.4 and 159.6 ± 15.4 pg/ml, respectively, after intravitreal injection, and these values were significantly lower than the 160.2 ± 7.6 and 151.9 ± 2.3 pg/ml, respectively, recorded at baseline (*F* = 5.043, *P* = 0.03; *F* = 4.888, *P* = 0.03). Although the concentration of PLGF in the aqueous humour samples obtained from patients decreased from 30.9 ± 1.0 pg/ml (baseline) to 30.5 ± 1.1 pg/ml (post-injection), this difference was not statistically significant (*F* = 1.376, *P* = 0.25). The concentrations of VEGF-A, TGF-β1, and PLGF in the aqueous humour of NVG patients were positively correlated with iris neovascularization at baseline (r = 0.919, *P* = 0.000; r = 0.923, P = 0.000; and r = 0.925, P = 0.000, respectively; See Table 3).

**Table 3:** The concentration of VEGF-A, PLGF, and TGF-β1 (pg/ml)

	time		*F*	*P*
	pre-injection	post-injection		
VEGF-A^b^	168.8 ± 13.4	160.2 ± 7.6 ^a^	5.043	0.03
PLGF^b^	30.9 ± 1.0	30.5 ± 1.1	1.376	0.25
TGF-β1^b^	159.6 ± 15.4	151.9 ± 2.3 ^a^	4.888	0.03

Compared with pre-injection, ^a^*P* < 0.05.^b^:ANOVA, LSD -*t* test

**Fig. 1:**
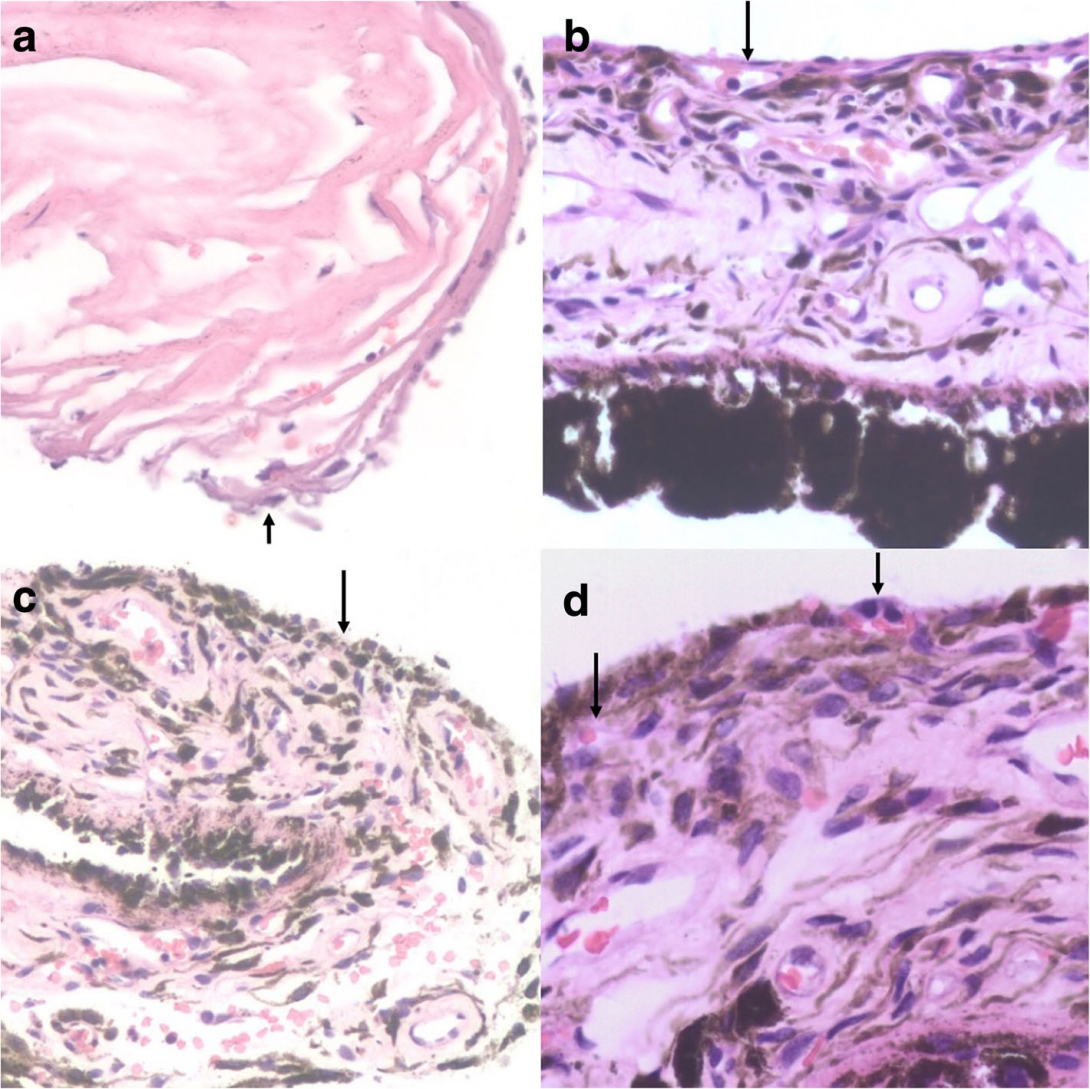
Light microscopy of the trabecular meshwork (**a**) and an iris treated with (**c**, **d**) and without (**b**) intravitreal conbercept, **a**, **b**, **c**, **d**: HE × 200

Under light microscopy, trabecular meshwork was observed in 14 of the 26 eyes that received intravitreal injection. We also noted that the trabecular band became thinner and had an irregular shape and that the endothelial cells at the surface of the trabecular band disappeared; the gap between the trabecular meshworks narrowed or the trabecular band linked together; small numbers of melanin granules were observed among the trabecular bands; a small number of blood cells surrounded adhered surgical specimens (this was unrelated to treatment); and no trabecular tissue was found in 12 eyes (this occurrence was associated with tissue not being cut intraoperatively or being embedded in the wrong direction; if, for example, the tissue was cut too far forward, the risk of bleeding, inflammation and ciliary body detachment increased) (Fig. 1a arrow).

Under light microscopy, in the specimens without intravitreal conbercept treatment, the thicknesses of the fibrous vascular membranes on the surface of the iris were different and many tiny new vessels were observed on the iris stroma (Fig. 1b arrow). The neovascularization of the iris surface significantly had regressed, but vessel blur remained (Fig. 1c, arrow). Small, thin-walled vessels were present in the anterior boundary layer and anterior stroma (Fig. 1d arrow) in specimens that received intravitreal injection. Iris stroma atrophy and disordered pigment cells in the stroma both increased, suggesting synechia, which is related to high IOP and the long-term application of hypotensive drugs [9].

**Fig. 2:**
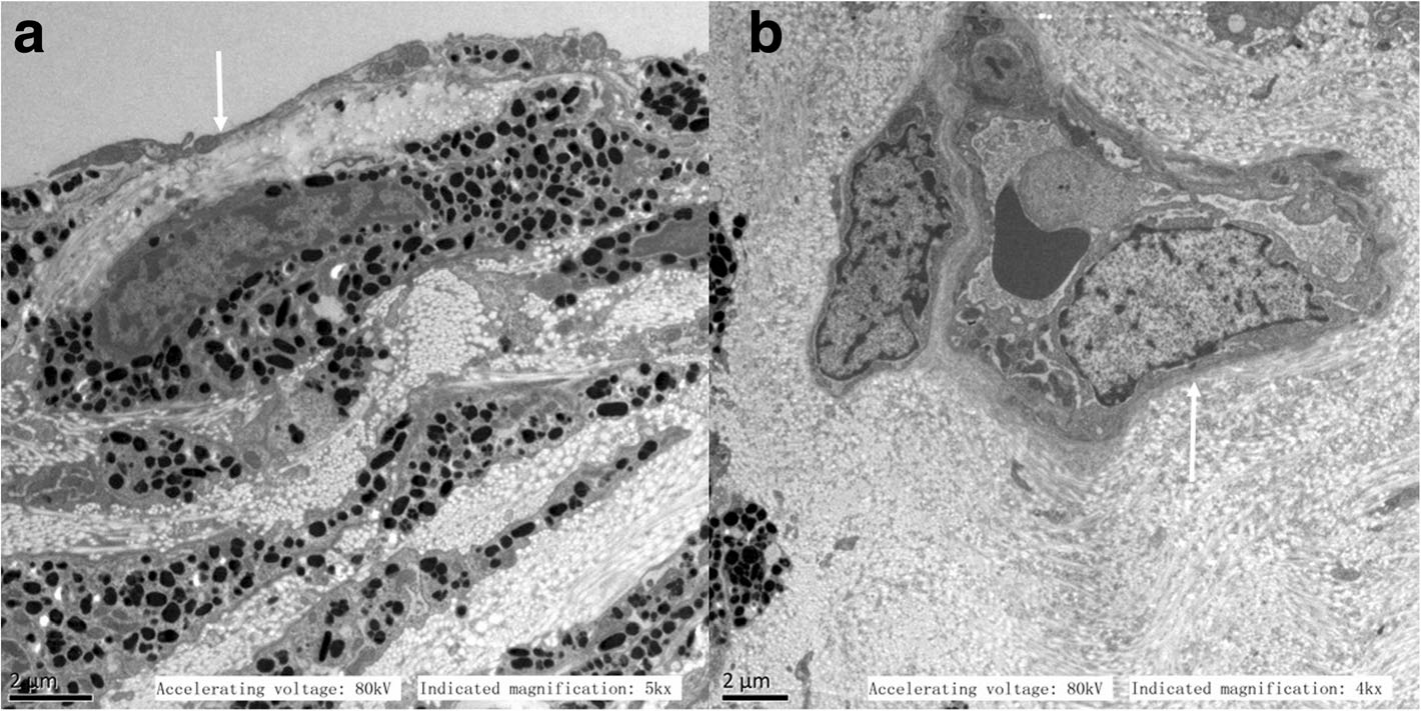
Transmission electron microscopy of an iris treated with intravitreal injection of conbercept. **a**: × 5000, bar = 2 μm; **b**: × 4000, bar = 2 μm

Under transmission electron microscopy, iris tissues were observed in 26 eyes that received intravitreal injection, and the results showed that there was no obvious neovascularization on the surface of the iris (Fig. 2a, arrow). Some neovascularization persisted in the iris stroma, and some of the vascular lumens had become narrowed (Fig. 2b, arrow) or atresic. The basement membrane of the blood vessel was incomplete, the material in the lumen, which included red blood cells, had disappeared, and the vascular endothelial cells had degenerated (Fig. 2b, arrow).

## Discussion

NVG is a serious and refractory type of glaucoma with poor prognosis, and the pathogenesis of NVG has not been fully clarified [10–12]. Research has shown that the occurrence and development of angiogenesis and NVG are closely related to various cytokines, such as VEGF, TGF-β1, interleukin-6, fibroblast growth factor, and platelet-derived growth factor [13–16]. The members of the VEGF family are VEGF-A (also referred to as VEGF), VEGF-B, VEGF-C, VEGF-D and PLGF, and among them, VEGF-A and PLGF have been shown to be associated with angiogenesis [13].

VEGF is released by retinal pigment epithelial cells in retinal ischaemia, and patients with NVG and its primary diseases have significantly elevated concentrations of VEGF in their aqueous humour and vitreous [17]. VEGF combines with two of its receptors, VEGFR-1 and -2, leading to vascular epithelial cell proliferation and migration and enhanced vascular permeability followed by vascular dilation and reconstruction. When VEGF is upregulated, it can spread through the aqueous humour to the anterior segment of the eye, resulting in iris and anterior chamber angle neovascularization. Fibrovascular membranes are then created, followed by synechia of the peripheral iris and trabecular meshwork, which can inhibit aqueous flow, leading to an increase in IOP [18–20].

Filtering surgery is the most effective method for the treatment of glaucoma [21]; however, in NVG patients, immediate surgery is associated with unfavourable outcomes, such as hyphema, filter bubble adhesion and choroidal detachment due to neovascularization in the iris and anterior chamber angle [22, 23]. Horsely et al. showed that the administration of intravitreal bevacizumab before trabeculectomy regressed iris neovascularization and reduced intraoperative and postoperative hyphema [24].

Anti-VEGF agents inhibit endothelial cell proliferation and angiogenesis by competitively inhibiting the ability of VEGF to bind to its receptors [25]. Common anti-VEGF agents include pegaptanib, bevacizumab and ranibizumab. Pegaptanib specifically combines with VEGF-165, while bevacizumab and ranibizumab combine with all subtypes of VEGF-A [26]. Another new agent is conbercept, which is a fusion protein derived from the extra-cellular domains of VEGFR-1 and -2 and the Fc portion of immunoglobulin G1. Conbercept shows high affinity for all subtypes of VEGF-A, VEGF-B and PLGF [5].

Surface iris neovascularization is derived from the anterior chamber angle and stroma, but Yosuke et al. showed that bevacizumab exerted different effects on the surface of the iris and stroma [27]. In our study, histopathological findings suggested that treatment caused neovascularization to regress, caused damage to neovascularized structures and reduced vascular permeability. Wakabayashi et al. showed that the time required for the complete regression of neovascularization was 1 day to 1 week after intravitreous bevacizumab [28]; however, long-term high IOP will irreversibly damage the optic nerve. Therefore, filtration surgery should be performed as soon as possible to reduce IOP and save visual function. The remaining atresia or cavity neovascularization of the iris stroma will then be reconstructed if the retinal ischaemia is not improved by the timely administration of anti-VEGF agents (8 to 10 weeks), making PRP necessary [29].

Hence, to reduce IOP and improve retinal ischaemia, neovascularization should be regressed with intravitreal anti-VEGF agents first before trabeculectomy combined with PRP is performed [30]. Studies have shown that the use of trabeculectomy and PRP followed by intravitreal injection of bevacizumab or ranibizumab is a safe and effective method for treating NVG that produces fewer postoperative complications [31–33].

Oshima et al. studied intravitreal bevacizumab, and after 2 months of follow-up, 29% of the cases had recurrent iris neovascularization [34]. Gheith et al. found that at 3 months and 5 months after treatment with intravitreal bevacizumab, iris neovascularization recurred [35]. Lin Zhaobin et al. found that 6 patients (27.3%) had recurrent iris neovascularization at 4 months after intravitreal bevacizumab treatment [36]. The recurrence of iris neovascularization may be related to the timeliness of PRP and treatment with anti-VEGF agents or that a portion of PRP energy was absorbed by the muddy lens or vitreous. In our study, patients with recurrent iris neovascularization received PRP, anti-glaucoma drugs and TCP, and neovascularization then partially regressed and IOP decreased.

In our study, IOP was significantly lower at the last visit than at baseline (*P* < 0.05), consistent with the results of Silva et al. and Lin Zhaobin et al. [29, 36]. Satoko et al. showed that in NVG patients, high IOP and poor angle function at baseline were associated with uncontrolled IOP postoperatively [37]. In our study, the baseline IOP was higher than 60 mmHg in 19 eyes, in which exudated depigmentation occurred, causing serious postoperative pigment membrane reactions. The trabecular meshwork and filtration path were obstructed and the angles completely closed in these patients, making IOP difficult to control. Four eyes received TCP; 14 eyes were treated with anti-glaucoma medicine, filter bleb massage and other treatments; and 1 eye received trabeculectomy to reduce IOP.

In our study, after treatment with intravitreal anti-VEGF agents, the concentration of VEGF-A in the aqueous humour significantly decreased, but the neovascularization of the iris and chamber angle were incompletely regressed. Therefore, we speculate that other cytokines may be involved in angiogenesis in these eyes. PLGF has been shown to enhance angiogenesis by increasing vascular permeability, promoting the inflammatory response and increasing VEGF-A activity. Therefore, PLGF may have a synergistic effect with VEGF-A during the development of NVG [12]. TGF-β1 plays an important role in cell growth, differentiation, apoptosis and immune regulation in addition to embryonic development, wound repair and other biological activities. Previous studies confirmed that the concentrations of TGF-β1 were significantly elevated in NVG patients and that TGF-β1 influenced the trabecular meshwork, resulting in decreased aqueous humour outflow and increased IOP, suggesting that TGF-β1 could play a role in the development of NVG [38, 39]. NVG may therefore be the result of angiogenesis, an immune response and inflammatory reactions. Anti-PLGF and anti-TGF-β1 treatment may be effective in NVG.

## Conclusions

Intravitreal injection of conbercept combined with trabeculectomy and PRP regressed iris neovascularization, reduced intraoperative and postoperative complications, and effectively controlled IOP and preserved visual function. In our study, two limitations were the small number of cases and the short follow-up time. Therefore, a long-term, larger sample study is needed to determine the efficacy and safety of conbercept for the treatment of NVG and provide new therapeutic targets for NVG.

## Additional file

Additional file 1: Raw data. (XLS 42 kb)

## Data Availability

All data generated or analysed during this study are included in this published article and its Additional file 1.

## Abbreviations

5-FU: 5-Fluorouracil
BCVA: Best-corrected visual acuity
CRVO: Central retinal vein occlusion
DR: Diabetic retinopathy
ELISA: Enzyme-linked immunosorbent assay
IOP: Intraocular pressure
LogMAR: Logarithm of the minimum angle of resolution
MMC: Mitomycin C
NVG: Neovascular glaucoma
PLGF: Placenta growth factor
PRP: Panretinal photocoagulation
TGF-β1: Transforming growth factor-β1
VEGF: Vascular endothelial growth factor
